# Science CommuniCa^2+^tion Developing Scientific Literacy on Calcium: The Involvement of CRAC Currents in Human Health and Disease

**DOI:** 10.3390/cells11111849

**Published:** 2022-06-05

**Authors:** Christina Humer, Sascha Berlansky, Herwig Grabmayr, Matthias Sallinger, Andreas Bernhard, Marc Fahrner, Irene Frischauf

**Affiliations:** Life Science Center, Johannes Kepler University Linz, Gruberstrasse 40, 4020 Linz, Austria; christina.humer_1@jku.at (C.H.); sascha.berlansky@jku.at (S.B.); herwig.grabmayr@jku.at (H.G.); matthias.sallinger@jku.at (M.S.); andreasxbernhard@gmail.com (A.B.); marc.fahrner@jku.at (M.F.)

**Keywords:** science communication, science literacy, calcium, store-operated channels, STIM, Orai, disease, cancer, therapeutic target

## Abstract

All human life starts with a calcium (Ca^2+^) wave. This ion regulates a plethora of cellular functions ranging from fertilisation and birth to development and cell death. A sophisticated system is responsible for maintaining the essential, tight concentration of calcium within cells. Intricate components of this Ca^2+^ network are store-operated calcium channels in the cells’ membrane. The best-characterised store-operated channel is the Ca^2+^ release-activated Ca^2+^ (CRAC) channel. Currents through CRAC channels are critically dependent on the correct function of two proteins: STIM1 and Orai1. A disruption of the precise mechanism of Ca^2+^ entry through CRAC channels can lead to defects and in turn to severe impacts on our health. Mutations in either STIM1 or Orai1 proteins can have consequences on our immune cells, the cardiac and nervous system, the hormonal balance, muscle function, and many more. There is solid evidence that altered Ca^2+^ signalling through CRAC channels is involved in the hallmarks of cancer development: uncontrolled cell growth, resistance to cell death, migration, invasion, and metastasis. In this work we highlight the importance of Ca^2+^ and its role in human health and disease with focus on CRAC channels.

## 1. Aim of This Work and the Importance of Calcium in Life

Society’s relationship with natural science is at a critical level. Never have the issues affecting science been more demanding and challenging, the public more interested, or the opportunities more apparent. However, public confidence in scientific advice has been shaken within the last 2 years of the COVID-19 pandemic. The progress of biotechnology seems to be far ahead of the awareness and approval of many people. We are now in a climate of deep concern due to public mistrust, unease, and occasional hostility. Since the start of the prevailing pandemic in 2020, science has made immense progress, especially in the design of a new era of vaccines. Unfortunately, to date, scientific progress and its knowledge are still out of reach for the majority of people. This leads to unwarranted doubts as well as disbelief in science and scientific facts. We believe this is primarily due to a misunderstanding of science amongst the lay population, especially in a time when more and more scientific publications are becoming freely accessible. In our opinion, the scientific community is missing interpersonal and clear communication skills. With this review, we thus emphasise communication of our scientific knowledge on Ca^2+^, with special emphasis on CRAC channels, to the broad, interested masses in order to give people better insight into science and possibly also to reduce their scepticism of new scientific findings. Thus, it is important to know—all human life starts with a calcium wave.

Calcium (Ca^2+^) is a major element of human life. It is the first signal in a fertilised egg, which occurs when the envelope of a female oocyte is breached by a male sperm cell [1]. Later in life and through a balanced diet, Ca^2+^ is absorbed mainly through dairy products and green leafy vegetables [2]. Ca^2+^ as a vital mineral is quantitatively the most important in the human body. The main amount (roughly 99%) is stored in bones and teeth, but the Ca^2+^ ion fulfils far more important roles in humans. Ca^2+^ serves a courier role inside our cells (as the so-called intracellular “second messenger”), meaning its concentration within cells is changed in response to an outside (extracellular) signal. This extracellular signal is called “first messenger” or “ligand”. Once its messenger-function is activated, Ca^2+^ holds remarkable roles important for fertility, birth, development, muscle contraction, and the function and regulation of cells and tissues; it plays important roles within our immune system and is associated with viral life cycles [3,4,5,6,7,8,9]. 

**Figure 1:**
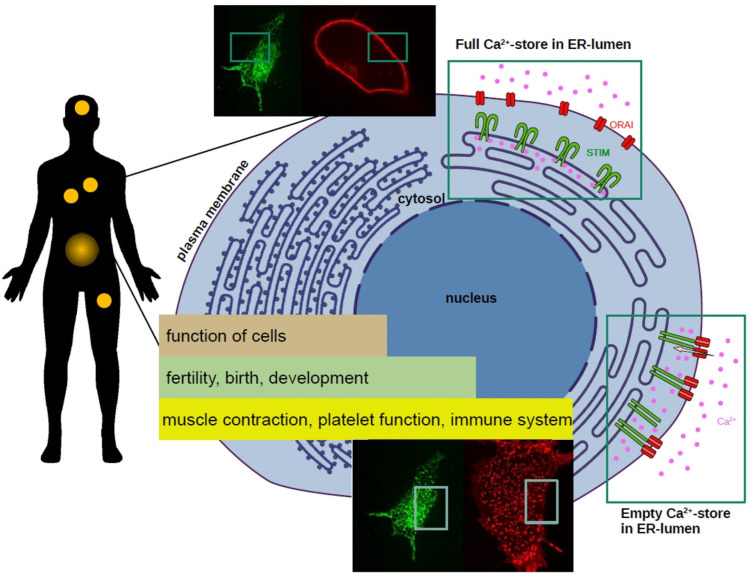
Calcium function from whole organism to single cell level. (**Left**): Biological processes and systems that depend on calcium. Yellow circles schematically mark sites affected by calcium-related diseases within the human body such as the brain, lungs, heart, muscles, and the immune system. (**Right**—middle lane): Scheme of a human cell. At resting conditions, calcium (purple) is stored within the ER. At this stage, calcium is bound to STIM1 proteins (green) which are in an inactive state. As soon as calcium stores are emptied, STIM1 loses the bound calcium, elongates, and communicates with Orai1 in the plasma membrane (red). Subsequently, calcium ions enter the cell from the extracellular milieu, trigger calcium-dependent responses, and the ER calcium stores are refilled as well. (**Right**—upper and lower lane): Live-cell fluorescent images of human embryonic kidney (HEK293) cells to visualise the localisation of STIM1 (green) and Orai1 (red) proteins within the cell. With full ER-calcium stores, both proteins are evenly distributed (upper lane). Upon emptying of the ER store, both proteins locate at sites called puncta where they interact (lower lane). (Image created by A.B.).

As Ca^2+^ regulates virtually all cellular functions and also has the potential to induce a cell’s death, it is mandatory to tightly maintain its balance (a process which is called “Ca^2+^ homeostasis”). A permanent increase of the Ca^2+^ concentration inside the cell (within the cytosol) is toxic and can lead to processes where our immune system attacks the body’s own cells (formally known as “autoimmune attacks”), inflammation of tissues, and other pathophysiological (“abnormal”) conditions [10,11,12]. Therefore, cells control their Ca^2+^ levels at all times. This monitoring system includes diverse transporters and ion channels within our cells to keep the concentration of Ca^2+^ in a temporally defined and tight range. Upon activation by a ligand, the cytosolic concentration of Ca^2+^ increases by 1000-fold through the release of Ca^2+^ from stores of the endoplasmic reticulum (ER—a network used to transport various substances around the cell). To maintain the high level of Ca^2+^, which is needed for cellular functions, the entry of extracellular Ca^2+^ is obligatory [8,13]. When needed, the cytosolic Ca^2+^ concentration can be increased in two different ways: either by releasing Ca^2+^ from ER stores within the cell, or by allowing Ca^2+^ entry through the cell membrane (also termed plasma membrane, PM) (Figure 1). As the internal stores of the ER have limited capacity, the main process of Ca^2+^ increase is through influx from the outside surroundings of the cell.

As mentioned above, Ca^2+^ can enter the cell via Ca^2+^ ion channels embedded in the plasma membrane. A primary route for this Ca^2+^ influx is through “store-operated channels”. These types of channels open in response to a signal of empty internal Ca^2+^ stores. This implies that there must be a communicated trigger signal from the cells’ interior to open up the “gate” in the membrane. The prototypic store-operated channel fulfilling these criteria is the so-called Ca^2+^ release-activated Ca^2+^ (CRAC) channel. In the normal physiological state, the CRAC channel transports Ca^2+^ into the cell just when needed. However, if this channel is changed in any way (due to inherited or acquired malfunctions, called “mutations”) this can have serious impacts on our health.

## 2. Calcium Uptake into the Cell—The CRAC Channel

The origin of all proteins lies in our genetic code, the deoxyribonucleic acid (DNA), which we inherit from our parents. Proteins are built according to instructions stored in certain areas of our DNA (the “genes”), which are essential for our survival. The CRAC channel system is based on the interaction of two proteins: one of them is partially located inside the ER, able to sense the Ca^2+^ concentration within. This protein is called stromal interaction molecule 1 (STIM1) [14,15]. The second protein involved is named Orai1, residing in the plasma membrane and acting as the actual Ca^2+^ channel [16,17] (Figure 1). One may be puzzled by the nomenclature of proteins in science, but it is quite easily explained: the person(s) who discover the protein get(s) to name it. For STIM1, the name stems from the identification of the first human form as cell adhesion molecule (necessary for attachment) in the stroma, the supporting tissue of an organ [18]. Even though the naming was later found to be inaccurate, it was still retained. Orai1 was named after the keepers of the gates of heaven in Greek mythology—the Horae [16]—at least here one can guess a figurative connection to a channel in the cell.

STIM1 traverses the membrane of the endoplasmic reticulum and includes a Ca^2+^ binding motif that can sense the stores’ Ca^2+^ concentration. As soon as the store loses Ca^2+^ for triggering a particular function within the cell, STIM1 redistributes into many local aggregates, known as “puncta” within the ER, which can be visualised with microscopic techniques (mainly this implies the labelling of proteins with a fluorescent tag—a “shiny” attachment needed for visualisation; Figure 1) [14,15]. These puncta are nothing other than hotspots of STIM1 aggregation to enable the interaction with Orai1 channels in the plasma membrane. Upon Ca^2+^ store depletion, STIM1 unfolds its cytosolic part (which is otherwise wrapped up close to the ER membrane) to bridge the distance to the usually far apart plasma membrane, allowing it to directly interact with Orai1 in the created puncta [19]. Once the interaction of the two proteins is established, the channel opens and allows Ca^2+^ ions to enter the cell and refill the internal stores.

Orai1 was discovered in 2006 in patients with a severe form of immunodeficiency (where the patients’ whole immune system is disrupted) [16]. There, an actual small error within Orai1 (called “point-mutation” where one single building block out of a total 301 of the protein is exchanged for another) leads to the loss of T-cell function; T-cells are the essential effector cells of our immune system (the name stems from their origin in the thymus gland, a specialised organ of the immune system). The T-cells lose their function (they are more or less paralysed) in response to a lack of Ca^2+^, which is not able to enter the cells due to the erroneous Orai1 channel, culminating in severe immune impairment. Physiologically healthy Orai1 proteins associate in groups of six, forming a so-called “hexamer”. This bunch of six proteins assembles in a circle with a hole in the centre that represents the actual funnel for Ca^2+^ entry (Figure 1). The structures of Orai1 proteins facing the cell’s interior hold several interaction sites for STIM1 [19,20] and the domains facing the outside of the cell can attract Ca^2+^ ions to allow for a facilitated Ca^2+^ flux upon channel opening [21]. In the case of the aforementioned immune disease, the Orai1 pore is permanently plugged so that no Ca^2+^ can enter the cell.

Such a two-component system as it exists for the CRAC channel is highly unusual in biology as it involves proteins from two different cellular compartments, the ER and PM, respectively. However, it is precisely this evolutionary feat that makes this system so important for humans. To date, mutated STIM1 and Orai1 proteins, and therefore an altered Ca^2+^ signal, have been associated with a variety of human diseases, underscoring their importance.

## 3. Calcium and Its Role in Human Diseases

The complex pattern of Ca^2+^ signalling is highly coordinated in space and time. One can easily believe that a disruption of these precise mechanisms can lead to defects [22,23]. The relationship of CRAC channels with human diseases first became obvious from studies in the 1990s. There, defects in CRAC channel function and subsequent Ca^2+^ entry in patients with a severe form of immunodeficiency (severe combined immunodeficiency, SCID) were reported to result in fatal infections with bacterial and viral pathogens [24,25,26,27]. At that time, the genes mutated in the immunodeficient patients were not known until the discovery of Orai1 proteins in 2006 [16,17,28]. Back then, it was shown that one single mutation within Orai1 leads to this loss-of-function (LoF) phenotype in SCID patients mentioned before. LoF implies that Orai1 loses its actual purpose and is no longer able to conduct Ca^2+^ into the cell when needed. This phenomenon, also called channelopathy (a pathological state that is caused by defects in a channel), has serious implications for our immune cells: without proper Ca^2+^ homeostasis, they simply are paralysed. The occurring syndrome in patients is characterised by recurrent and chronic infections, immune cells attacking our own cells (“autoimmunity”), a lower-than-normal number of platelets in the blood (“thrombocytopenia”), a state of low muscle tone (“muscular hypotonia”), problems with the skin (“ectodermal dysplasia”, describing inherited conditions that cause defects in structures emerging from the ectoderm of the embryo such as hair, teeth, and skin), and the inability to sweat (“anhidrosis”) [29]. 

To examine pathological functions of genes in living organisms, scientists usually make use of model organisms to study specific mutations in proteins. For an animal to become a model organism in science, it must have several characteristics: a comparatively small size and it should be easy to keep and reproduce quickly. Only under these conditions can scientific results be obtained with reasonable effort and in a relatively short time. Mice usually serve as prototypic model systems—hardly any mammalian organism has been studied as intensely as the mouse—its genome (the collection of all genes) has been completely decoded and 99% of the genes in the mouse genome have a similar form in humans [30]. The use of mice has shown us that Orai1 and STIM1 proteins play important roles in the network that controls our heartbeat (“cardiac conduction”), within the nervous system (“nociception”, a process by which noxious stimulation is communicated through the nervous system), neurodegenerative diseases (affecting the signal transmission of nerve cells, as in Alzheimer’s or Parkinson’s disease), cardiopulmonary disorders (related to heart and lungs), and ischemic stroke (within the brain) [31,32,33,34,35]. In principle, results in mice cannot be completely extrapolated, but they provide clues as to what effect these mutations might have in humans.

Apart from LoF mutations that can occur in proteins, alterations with the consequence of higher activity of the protein may also lead to disease. In this regard, a gain-of-function (GoF) mutation in STIM1 is associated with the so-called Stormorken syndrome (named after its discoverer) [36]. This GoF mutation induces disproportionate Ca^2+^ entry into the cell and patients suffer from an excessive contraction of the pupil of the eye (“miosis”), thrombocytopenia, intellectual disability, muscle fatigue, the absence of normal spleen function (“asplenia”), and they exhibit a condition where the skin becomes dry and horny (“ichthyosis”). A related disease is tubular aggregate myopathy (TAM, affecting primarily the skeletal muscles), a similar multisystemic disease, varying in disease severity and age of onset [37,38,39]. Variations in Orai1 have also been discovered and linked to Kawasaki disease [40], a systemic inflammation of blood vessels (“vasculitis”) which predominantly affects infants and children.

An essential role of CRAC channel proteins has also been proven for cells of the innate and adaptive immune system in humans [41,42]. The term innate immune system is understood to mean cells that are already present at birth, such as macrophages, neutrophils, and dendritic cells. Macrophages are phagocytes (they “eat” other cells), highly specialised in the removal of dying/dead cells and cell remains (“debris”). Neutrophils are white blood cells that help our body fight infections such as bacteria or viruses (foreign invaders are called “antigens” or “pathogens”). Dendritic cells serve as a link between the innate and adaptive immune system as they can capture, process, and present antigens to more specialised cells which afterwards respond to this antigen with effector functions (to directly fight antigens) or the creation of highly specific antibodies. Antibodies are the “heroes” of our immune system: after a production phase of approximately 10 days, they can accurately detect and erase foreign antigens. The adaptive immune system comprises everything we have “learned” during our lifetime by experience: specialised cells have adapted to specific antigens after encountering them the first time. These cells are mainly T-cells (also called T-lymphocytes, derived from the thymus) and B-cells (also called B-lymphocytes, derived from the bone marrow; the only cells that can produce antibodies). Since the immune system is constantly protecting our body from external attacks, it is easy to understand the need to maintain the balance of Ca^2+^—a task accomplished by STIM and Orai proteins. 

Ca^2+^ is also well-established to be important for the transmission of signals via nerve cells (neurons) and for the contraction of heart muscles (cardiomyocytes). There is evidence that Ca^2+^ is involved in neurodegenerative diseases such as Alzheimer’s, Huntington’s, and Parkinson’s disease [35]. In addition, the latest findings point to an association of CRAC channel proteins in the nervous system and pain [34]. Ca^2+^ signals are furthermore known to play an essential role in infection with diverse viruses. Therefore, Ca^2+^ is important for virus-host cell interaction [7], viral replication, and release [6,9,43]. Viruses have the ability to dysregulate Ca^2+^ dynamics within a cell [44]. During the ongoing coronavirus disease 2019 (COVID-19) pandemic, it has been shown that Ca^2+^ can bind to severe acute respiratory syndrome coronavirus 2, the COVID-19-causing virus (SARS-CoV-2), leading to a facilitated viral entry into human cells [45,46]. This makes Ca^2+^ channels a feasible drug target site to prevent COVID-19; this has already been successfully proven to be effective against the common flu (influenza A) or ebolavirus-triggered disease [46]. Yet not all Ca^2+^-dependent mechanisms of SARS-CoV-2 have been fully deciphered and are still under investigation [43].

Apart from viral infections, disturbances in Ca^2+^ homeostasis are also linked to cancer. Cancer as the leading cause of death globally comprises more than 100 different types, affecting any part of the body in both sexes [47]. Cancer caused nearly 10 million deaths worldwide in 2020. The most common cancer types in women are breast (25.8%), colorectal (9.9%), lung (8.8%), and cervix (6.9%). Whereas lung (15.4%), prostate (15.1%), and colorectal (11.4%) cancers are the most common among men [48]. Pathophysiological features of cancer are known as cancer hallmarks [49,50]. Of these hallmarks, three are critically dependent on Ca^2+^: (i) enhanced growth (“proliferation”) of cells (meaning their insensitivity to anti-growth signals and the fact that they show limitless reproduction), (ii) resistance to programmed cell death (a mechanism by which the cell is destroyed by autologous mechanisms, also called “apoptosis”), and (iii) migration, invasion, and metastasis (the movement, intrusion, and growth at other body sites via vascularisation and angiogenesis) of cancer cells [51,52,53]. Over the last years, many research groups have investigated the role of Orai and STIM proteins in cancer development [54,55]. The first evidence found stems from the 1980s when Ca^2+^ channel blockers were identified as inhibitors of cancer growth [56,57,58]. Meanwhile, it has been discovered that Ca^2+^ activates several key signalling pathways and transcription factors (needed for the production of proteins) that control cell proliferation and the cell cycle in cancer cells [59,60,61,62]. 

Most major carcinomas develop from epithelial origin, meaning cells that line hollow organs and glands. In epithelial cells, the main pathway for Ca^2+^ entry is through store-operated Ca^2+^ channels, especially CRAC channels [63,64]. To what extent Orai1 proteins have the potential to cause cancerous remodelling is dependent on the cancer type and the particular context [31]. Furthermore, Ca^2+^ is essential for human brain tumour glioblastoma invasion [65] and for invasive stimuli in breast cancer [66,67,68]. Melanoma cells are dependent on Orai1 which plays a central role in cell migration and proliferation [69,70]. Moreover, in colon and breast cancer, Orai1 plays a fundamental role in invasion and metastasis [71,72]. It has been shown for prostate cancer that remodelling of Ca^2+^ homeostasis leads to resistance to apoptosis [73,74], due to a decreased level of Orai1 proteins expressed in the cell. Not only Orai1 but also STIM1 can be a cause of cancer development. It has been demonstrated that STIM1 proteins play major roles in glioblastomas, cervix, breast, lung, liver, melanoma, and colon carcinomas [66,69,75,76,77,78,79]. Within these cancers one can find higher levels of STIM1 proteins than in healthy tissues. 

Unmentioned so far, there are other very similar proteins besides Orai1 and STIM1. The information for the synthesis of further Orai and STIM proteins is encoded within the DNA of humans; these are referred to as Orai2, Orai3, and STIM2. These “relatives” are scientifically termed “homologs” and, much like siblings, show common and differentiating features and functions. All three Orai proteins can, for instance, be activated by STIM1 if Ca^2+^ is low inside the ER, but they differ at key sites that interact with STIM1. STIM2 is more sensitive to Ca^2+^ but is weaker in activating Orai. Not all tissues and cell types need all of the homologs, implying that there are differences in the requirement of their expression. Yet the interplay of STIM1 and STIM2 with the Orai proteins shapes the Ca^2+^ signal and is important in deciding for a specific cellular response and not the other. Moreover, STIM2 and Orai2/3 are relevant to cancerous processes, whereby Orai2 was, for instance, reported to promote migration and metastasis in gastric cancer [80,81] and is important for cell cycle progression of breast cancer cells [82]. STIM2 is relevant in leukaemia [83], while Orai3 is associated with the migration of breast cancer cells [84] and is linked to pancreatic tumour growth [85]. Overall, a tremendous amount of research has been performed on the STIM1 and Orai1 isoforms over the past 17 years. These two proteins seem to play a major role in the vast majority of cell types regarding the CRAC channel system. Accordingly, less is found in the literature about the other isoforms (STIM2 and Orai2/Orai3). It is possible that mixed forms are present, whereby the combination of STIM1/2 or Orai1/2/3 builds the CRAC channel. However, the extent to which this is physiologically relevant is still poorly understood and requires further investigation.

## 4. Outlook

As outlined in this work, Ca^2+^ plays a major role in human health and disease. To date, there are no drugs currently being used clinically that target the Ca^2+^ signalling machinery, although several pharmaceutical companies are working on it. In order to develop new drugs, the molecular structures of the proteins involved must first be deciphered [86]. This is known under the term “basic research”. Only then can the focus turn to applied research to develop specific substances targeting the Ca^2+^ signalling machinery in cells. Understanding the structure of CRAC channels, meaning STIM and Orai proteins, will improve the prospects for developing novel and relatively untapped therapeutic agents which aim at combating the growing list of human diseases associated with aberrant store-operated Ca^2+^ influx in humans, including cancer and COVID-19.

Communicating the current state of science to society is often a difficult task. We hope this paper is a first step in helping people without a background in biology understand the important role calcium plays in humans and the importance of research being conducted on this topic. No one benefits from scientific progress if it is not understood and broadly accepted.

## References

[1] Santella L., Lim D., Moccia F. (2004). Calcium and fertilization: The beginning of life. Trends Biochem. Sci..

[2] United Kingdom National Health Service Department of Health and social care UK Vitamins and minerals—Calcium.

[3] Berridge M.J., Lipp P., Bootman M.D. (2000). The versatility and universality of calcium signalling. Nat. Rev. Mol. Cell Biol..

[4] Carafoli E. (2002). Calcium signaling: A tale for all seasons. Proc. Natl. Acad. Sci. USA.

[5] Clapham D.E. (2007). Calcium Signaling. Cell.

[6] Brini M., Ottolini D., Calì T., Carafoli E., Sigel A., Sigel H., Sigel R.K.O. (2013). Calcium in Health and Disease. Interrelations between Essential Metal Ions and Human Diseases.

[7] Nieto-Torres J.L., Verdiá-Báguena C., Jimenez-Guardeno J.M.J., Regla-Nava J.A., Castaño-Rodriguez C., Fernandez-Delgado R., Torres J., Aguilella V.M., Enjuanes L. (2015). Severe acute respiratory syndrome coronavirus E protein transports calcium ions and activates the NLRP3 inflammasome. Virology.

[8] DeHaven W.I., Smyth J.T., Boyles R.R., Putney J.W. (2007). Calcium inhibition and calcium potentiation of Orai1, Orai2, and Orai3 calcium release-activated calcium channels. J. Biol. Chem..

[9] Murakami T., Ockinger J., Yu J., Byles V., McColl A., Hofer A.M., Horng T. (2012). Critical role for calcium mobilization in activation of the NLRP3 inflammasome. Proc. Natl. Acad. Sci. USA.

[10] Petersen O.H., Sutton R. (2006). Ca^2+^ signalling and pancreatitis: Effects of alcohol, bile and coffee. Trends Pharmacol. Sci..

[11] Petersen O.H., Tepikin A.V. (2008). Polarized Calcium Signaling in Exocrine Gland Cells. Annu. Rev. Physiol..

[12] Masuyama R., Vriens J., Voets T., Karashima Y., Owsianik G., Vennekens R., Lieben L., Torrekens S., Moermans K., Bosch A.V. (2008). TRPV4-Mediated Calcium Influx Regulates Terminal Differentiation of Osteoclasts. Cell Metab..

[13] Lewis R.S. (2007). The molecular choreography of a store-operated calcium channel. Nature.

[14] Roos J., Digregorio P.J., Yeromin A.V., Ohlsen K., Lioudyno M., Zhang S., Safrina O., Kozak J.A., Wagner S.L., Cahalan M.D. (2005). STIM1, an essential and conserved component of store-operated Ca^2+^ channel function. J. Cell Biol..

[15] Liou J., Kim M.L., Do Heo W., Jones J.T., Myers J.W., Ferrell J.E., Meyer T. (2005). STIM Is a Ca^2+^ Sensor Essential for Ca^2+^-Store-Depletion-Triggered Ca^2+^ Influx. Curr. Biol..

[16] Feske S., Gwack Y., Prakriya M., Srikanth S., Puppel S.-H., Tanasa B., Hogan P.G., Lewis R.S., Daly M., Rao A. (2006). A mutation in Orai1 causes immune deficiency by abrogating CRAC channel function. Nature.

[17] Vig M., Peinelt C., Beck A., Koomoa D.L., Rabah D., Koblan-Huberson M., Kraft S., Turner H., Fleig A., Penner R. (2006). CRACM1 is a plasma membrane protein essential for store-operated Ca^2+^ entry. Science.

[18] Oritani K., Kincade P.W. (1996). Identification of stromal cell products that interact with pre-B cells. J. Cell Biol..

[19] Muik M., Frischauf I., Derler I., Fahrner M., Bergsmann J., Eder P., Schindl R., Hesch C., Polzinger B., Fritsch R. (2008). Dynamic Coupling of the Putative Coiled-coil Domain of ORAI1 with STIM1 Mediates ORAI1 Channel Activation. J. Biol. Chem..

[20] Fahrner M., Derler I., Jardin I., Romanin C. (2013). The STIM1/Orai signaling machinery. Channels.

[21] Frischauf I., Zayats V., Deix M., Hochreiter A., Jardin I., Muik M., Lackner B., Svobodová B., Pammer T., Litviňuková M. (2015). A calcium-accumulating region, CAR, in the channel Orai1 enhances Ca^2+^ permeation and SOCE-induced gene transcription. Sci. Signal..

[22] Parekh A.B. (2019). Calcium signalling in health and disease. Semin. Cell Dev. Biol..

[23] Feske S. (2019). CRAC channels and disease – From human CRAC channelopathies and animal models to novel drugs. Cell Calcium.

[24] Feske S., Giltnane J., Dolmetsch R., Staudt L.M., Rao A. (2001). Gene regulation mediated by calcium signals in T lymphocytes. Nat. Immunol..

[25] Feske S., Müller J.M., Graf D., Kroczek R.A., Dräger R., Niemeyer C., Baeuerle P.A., Peter H.-H., Schlesier M. (1996). Severe combined immunodeficiency due to defective binding of the nuclear factor of activated T cells in T lymphocytes of two male siblings. Eur. J. Immunol..

[26] Le Deist F., Hivroz C., Partiseti M., Thomas C., Buc H.A., Oleastro M., Belohradsky B., Choquet D., Fischer A. (1995). A pri-mary T-cell immunodeficiency associated with defective transmembrane calcium influx. Blood.

[27] Partiseti M., Le Deist F., Hivroz C., Fischer A., Korn H., Choquet D. (1994). The calcium current activated by T cell receptor and store depletion in human lymphocytes is absent in a primary immunodeficiency. J. Biol. Chem..

[28] Zhang S.L., Yeromin A.V., Zhang X.H.-F., Yu Y., Safrina O., Penna A., Roos J., Stauderman K.A., Cahalan M.D. (2006). Genome-wide RNAi screen of Ca^2+^ influx identifies genes that regulate Ca^2+^ release-activated Ca^2+^ channel activity. Proc. Natl. Acad. Sci. USA.

[29] Vaeth M., Feske S. (2018). Ion channelopathies of the immune system. Curr. Opin. Immunol..

[30] Vandamme T.F. (2014). Use of rodents as models of human diseases. J. Pharm. Bioallied Sci..

[31] Chalmers S.B., Monteith G.R. (2018). ORAI channels and cancer. Cell Calcium.

[32] Johnson M., Trebak M. (2019). ORAI channels in cellular remodeling of cardiorespiratory disease. Cell Calcium.

[33] Mammadova-Bach E., Nagy M., Heemskerk J.W., Nieswandt B., Braun A. (2018). Store-operated calcium entry in thrombosis and thrombo-inflammation. Cell Calcium.

[34] Mei Y., Barrett J.E., Hu H. (2018). Calcium release-activated calcium channels and pain. Cell Calcium.

[35] Wegierski T., Kuznicki J. (2018). Neuronal calcium signaling via store-operated channels in health and disease. Cell Calcium.

[36] Morin G., Bruechle N.O., Singh A.R., Knopp C., Jedraszak G., Elbracht M., Bremond-Gignac D., Hartmann K.A., Sevestre H., Deutz P. (2014). Gain-of-Function Mutation in STIM1 (P.R304W) Is Associated with Stormorken Syndrome. Hum. Mutat..

[37] Böhm J., Laporte J. (2018). Gain-of-function mutations in STIM1 and ORAI1 causing tubular aggregate myopathy and Stormorken syndrome. Cell Calcium.

[38] Michelucci A., García-Castañeda M., Boncompagni S., Dirksen R.T. (2018). Role of STIM1/ORAI1-mediated store-operated Ca2+ entry in skeletal muscle physiology and disease. Cell Calcium.

[39] Endo Y., Noguchi S., Hara Y., Hayashi Y.K., Motomura K., Miyatake S., Murakami N., Tanaka S., Yamashita S., Kizu R. (2014). Dominant mutations in ORAI1 cause tubular aggregate myopathy with hypocalcemia via constitutive activation of store-operated Ca^2+^ channels. Hum. Mol. Genet..

[40] Onouchi Y., Fukazawa R., Yamamura K., Suzuki H., Kakimoto N., Suenaga T., Takeuchi T., Hamada H., Honda T., Yasukawa K. (2016). Variations in ORAI1 Gene Associated with Kawasaki Disease. PLoS ONE.

[41] Clemens R.A., Lowell C.A. (2019). CRAC channel regulation of innate immune cells in health and disease. Cell Calcium.

[42] Feske S. (2007). Calcium signalling in lymphocyte activation and disease. Nat. Rev. Immunol..

[43] Berlansky S., Sallinger M., Grabmayr H., Humer C., Bernhard A., Fahrner M., Frischauf I. (2022). Calcium Signals during SARS-CoV-2 Infection: Assessing the Potential of Emerging Therapies. Cells.

[44] Zhou Y., Xue S., Yang J.J., Kretsinger R.H., Uversky V.N., Permyakov E.A. (2013). Calcium and Viruses. Encyclopedia of Metalloproteins.

[45] Lai A.L., Freed J.H. (2021). SARS-CoV-2 Fusion Peptide has a Greater Membrane Perturbating Effect than SARS-CoV with Highly Specific Dependence on Ca^2+^. J. Mol. Biol..

[46] Nathan L., Lai A.L., Millet J.K., Straus M.R., Freed J.H., Whittaker G.R., Daniel S. (2019). Calcium Ions Directly Interact with the Ebola Virus Fusion Peptide To Promote Structure–Function Changes That Enhance Infection. ACS Infect. Dis..

[47] WHO Cancer. 3 February 2022.

[48] World Cancer Research Fund Global cancer statistics for the most common cancers in the world.

[49] Hanahan D., Weinberg R.A. (2000). The Hallmarks of Cancer. Cell.

[50] Tajada S., Villalobos C. (2020). Calcium Permeable Channels in Cancer Hallmarks. Front. Pharmacol..

[51] Prevarskaya N., Ouadid-Ahidouch H., Skryma R., Shuba Y. (2014). Remodelling of Ca^2+^ transport in cancer: How it contributes to cancer hallmarks?. Philos. Trans. R. Soc. Lond. B Biol. Sci..

[52] Monteith G.R., Prevarskaya N., Roberts-Thomson S.J. (2017). The calcium–cancer signalling nexus. Nat. Rev. Cancer.

[53] Prevarskaya N., Skryma R., Shuba Y. (2018). Ion Channels in Cancer: Are Cancer Hallmarks Oncochannelopathies?. Physiol. Rev..

[54] So C.L., Saunus J.M., Roberts-Thomson S.J., Monteith G.R. (2018). Calcium signalling and breast cancer. Semin. Cell Dev. Biol..

[55] Shuba Y. (2019). Ca^2+^ channel-forming ORAI proteins: Cancer foes or cancer allies?. Exp. Oncol..

[56] Batra S., Alenfall J. (1991). Effect of diverse categories of drugs on human colon tumour cell proliferation. Anticancer Res..

[57] Taylor J.M., Simpson R.U. (1992). Inhibition of cancer cell growth by calcium channel antagonists in the athymic mouse. Cancer Res..

[58] Lee S.C., Deutsch C., Beck W.T. (1988). Comparison of ion channels in multidrug-resistant and -sensitive human leukemic cells. Proc. Natl. Acad. Sci. USA.

[59] Dolmetsch R.E., Xu K., Lewis R.S. (1998). Calcium oscillations increase the efficiency and specificity of gene expression. Nature.

[60] Li W.-H., Llopis J., A Whitney M., Zlokarnik G., Tsien R.Y. (1998). Cell-permeant caged InsP3 ester shows that Ca^2+^ spike frequency can optimize gene expression. Nature.

[61] Walker S., Kupzig S., Bouyoucef D., Davies L.C., Tsuboi T., Bivona T.G., Cozier G., Lockyer P.J., Buckler A., Rutter G. (2004). Identification of a Ras GTPase-activating protein regulated by receptor-mediated Ca^2+^ oscillations. EMBO J..

[62] Liu J., Maller J.L. (2005). Calcium Elevation at Fertilization Coordinates Phosphorylation of XErp1/Emi2 by Plx1 and CaMK II to Release Metaphase Arrest by Cytostatic Factor. Curr. Biol..

[63] Wei J., Deng Y., Ye J., Luo Y., Weng J., He Q., Liu F., Li M., Liang R., Lin Y. (2021). Store-operated Ca^2+^ entry as a key oncogenic Ca^2+^ signaling driving tumor invasion-metastasis cascade and its translational potential. Cancer Lett..

[64] Bruce J.I.E., James A.D. (2020). Targeting the Calcium Signalling Machinery in Cancer. Cancers.

[65] Motiani R.K., Hyzinski-García M.C., Zhang X., Henkel M.M., Abdullaev I.F., Kuo Y.-H., Matrougui K., Mongin A.A., Trebak M. (2013). STIM1 and Orai1 mediate CRAC channel activity and are essential for human glioblastoma invasion. Pflügers Archiv-Eur. J. Physiol..

[66] McAndrew D., Grice D.M., Peters A.A., Davis F.M., Stewart T., Rice M., Smart C.E., Brown M.A., Kenny P.A., Roberts-Thomson S.J. (2011). ORAI1-Mediated Calcium Influx in Lactation and in Breast Cancer. Mol. Cancer Ther..

[67] Chamlali M., Rodat-Despoix L., Ouadid-Ahidouch H. (2021). Store-Independent Calcium Entry and Related Signaling Pathways in Breast Cancer. Genes.

[68] Zhou W., Pan H., Xia T., Xue J., Cheng L., Fan P., Zhang Y., Zhu W., Xue Y., Liu X. (2014). Up-regulation of S100A16 expression promotes epithelial-mesenchymal transition via Notch1 pathway in breast cancer. J. Biomed. Sci..

[69] Umemura M., Baljinnyam E., Feske S., De Lorenzo M.S., Xie L.-H., Feng X., Oda K., Makino A., Fujita T., Yokoyama U. (2014). Store-Operated Ca^2+^ Entry (SOCE) Regulates Melanoma Proliferation and Cell Migration. PLoS ONE.

[70] Stanisz H., Stark A., Kilch T., Schwarz E.C., Müller C.S., Peinelt C., Hoth M., Niemeyer B.A., Vogt T., Bogeski I. (2012). ORAI1 Ca^2+^ Channels Control Endothelin-1-Induced Mitogenesis and Melanogenesis in Primary Human Melanocytes. J. Investig. Dermatol..

[71] Gueguinou M., Crottès D., Chantôme A., Rapetti-Mauss R., Potier-Cartereau M., Clarysse L., Girault A., Fourbon Y., Jézéquel P., Guérin-Charbonnel C. (2017). The SigmaR1 chaperone drives breast and colorectal cancer cell migration by tuning SK3-dependent Ca^2+^ homeostasis. Oncogene.

[72] Leverrier-Penna S., Destaing O., Penna A. (2020). Insights and perspectives on calcium channel functions in the cockpit of cancerous space invaders. Cell Calcium.

[73] Skryma R., Mariot P., Bourhis X., Coppenolle F., Shuba Y., Abeele F.V., Legrand G., Humez S., Boilly B., Prevarskaya N. (2000). Store depletion and store-operated Ca^2+^ current in human prostate cancer LNCaP cells: Involvement in apoptosis. J. Physiol..

[74] Perrouin-Verbe M.A., Schoentgen N., Talagas M., Garlantezec R., Uguen A., Doucet L., Rosec S., Marcorelles P., Potier-Cartereau M., Vandier C. (2019). Overexpression of certain transient receptor potential and Orai channels in prostate cancer is associated with decreased risk of systemic recurrence after radical prostatectomy. Prostate.

[75] Motta F., Valera E., Lucio-Eterovic A., Queiroz R., Neder L., Scrideli C., Machado H., Carlotti-Junior C., Marie S., Tone L. (2008). Differential expression of E-cadherin gene in human neuroepithelial tumors. Genet. Mol. Res..

[76] Chen J., Yao Y., Gong C., Yu F., Su S., Chen J., Liu B., Deng H., Wang F., Lin L. (2011). CCL18 from Tumor-Associated Macrophages Promotes Breast Cancer Metastasis via PITPNM3. Cancer Cell.

[77] Li J., Koh W.-P., Jin A.-Z., Yuan J.-M., Yu M.C., Butler L.M. (2013). Calcium intake is not related to breast cancer risk among Singapore Chinese women. Int. J. Cancer.

[78] Yang B., Cao L., Liu B., McCaig C.D., Pu J. (2013). The Transition from Proliferation to Differentiation in Colorectal Cancer Is Regulated by the Calcium Activated Chloride Channel A1. PLoS ONE.

[79] Guan L., Song Y., Gao J., Gao J., Wang K. (2016). Inhibition of calcium-activated chloride channel ANO1 suppresses proliferation and induces apoptosis of epithelium originated cancer cells. Oncotarget.

[80] Wu S., Chen M., Huang J., Zhang F., Lv Z., Jia Y., Cui Y.-Z., Sun L.-Z., Wang Y., Tang Y. (2020). ORAI2 Promotes Gastric Cancer Tumorigenicity and Metastasis through PI3K/Akt Signaling and MAPK-Dependent Focal Adhesion Disassembly. Cancer Res..

[81] Motiani R.K., Zhang X., Harmon K.E., Keller R.S., Matrougui K., Bennett J.A., Trebak M. (2012). Orai3 is an estrogen receptor α-regulated Ca^2+^ channel that promotes tumorigenesis. FASEB J..

[82] Sanchez-Collado J., Lopez J.J., Cantonero C., Jardin I., Regodón S., Redondo P.C., Gordillo J., Smani T., Salido G.M., Rosado J.A. (2021). Orai2 Modulates Store-Operated Ca^2+^ Entry and Cell Cycle Progression in Breast Cancer Cells. Cancers.

[83] Fleur-Lominy S.S., Maus M., Vaeth M., Lange I., Zee I., Suh D., Liu C., Wu X., Tikhonova A., Aifantis I. (2018). STIM1 and STIM2 Mediate Cancer-Induced Inflammation in T Cell Acute Lymphoblastic Leukemia. Cell Rep..

[84] Chamlali M., Kouba S., Rodat-Despoix L., Todesca L.M., Pethö Z., Schwab A., Ouadid-Ahidouch H. (2021). Orai3 Calcium Channel Regulates Breast Cancer Cell Migration through Calcium-Dependent and -Independent Mechanisms. Cells.

[85] Dubois C., Kondratska K., Kondratskyi A., Morabito A., Mesilmany L., Farfariello V., Toillon R.-A., Gelus N.Z., Laurenge E., Abeele F.V. (2021). ORAI3 silencing alters cell proliferation and promotes mitotic catastrophe and apoptosis in pancreatic adenocarcinoma. Biochim. Biophys. Acta.

[86] Chang Y., Roy S., Pan Z. (2021). Store-Operated Calcium Channels as Drug Target in Gastroesophageal Cancers. Front. Pharmacol..

